# Identification of the prognostic effect of mitophagy-related genes in acute myeloid leukemia

**DOI:** 10.3389/fimmu.2025.1580597

**Published:** 2025-08-12

**Authors:** Fan Xuan, Wenyuan Zhu, Baoxi Zhang, Hui Zhao, Chaonan Li, Xiaoli Wu

**Affiliations:** ^1^ Department of Pediatrics, Hematology and Oncology, Second Hospital of Hebei Medical University, Shijiazhuang, China; ^2^ Graduate School, Hebei Medical University, Shijiazhuang, Hebei, China

**Keywords:** acute myeloid leukemia, mitophagy-related genes, biomarkers, prognostic effect, immune infiltration

## Abstract

**Background:**

Mitophagy has been implicated in the pathogenesis of acute myeloid leukemia (AML), yet its precise molecular mechanisms remain poorly understood. Understanding the roles of mitophagy-related genes (MRGs) may provide new insights into AML classification, prognosis, and therapeutic response.

**Methods:**

We analyzed 72 MRGs using three independent AML datasets (TCGA-LAML, GSE24395, and GSE146173). Consensus clustering based on MRG expression was used to identify AML molecular subtypes. Differentially expressed genes (DEGs) common to AML subtypes and GSE24395 were identified. Prognostic genes were screened using univariate Cox regression and least absolute shrinkage and selection operator (Lasso) regression analyses. A prognostic risk model was constructed and validated. Functional enrichment, immune infiltration, and drug sensitivity analyses were conducted to explore the biological relevance of the model. In addition, regulatory elements including microRNAs, lncRNAs, and transcription factors targeting model genes were predicted.

**Results:**

Twenty-six overlapping DEGs were identified between AML subtypes and GSE24395. Five MRG-associated genes (*ITGB2*, *VIP*, *PTK2*, *FHL2*, *BAG3*) were selected to construct a prognostic model that stratified patients into high- and low-risk groups with significantly different overall survival. Multivariate Cox analysis confirmed that risk score, age, and treatment status were independent prognostic indicators. Gene set enrichment analysis (GSEA) revealed 731 significantly enriched pathways, including mononuclear cell migration. Immune cell infiltration analysis showed a positive correlation between risk score and monocytes, and negative correlations with plasma B cells and activated mast cells. Drug sensitivity prediction identified 84 compounds with differential responses between risk groups. Regulatory network prediction highlighted hsa-miR-135b-5p, FTX, and SOX11 as potential upstream regulators of the prognostic genes.

**Conclusion:**

This study identified five mitophagy-related genes as prognostic biomarkers in AML and developed a robust risk model that correlates with survival outcome, immune infiltration, and drug sensitivity. These findings offer new insights into mitophagy-related mechanisms in AML and may guide personalized therapeutic strategies.

## 1 Introduction

Acute myeloid leukemia (AML), one of the most prevalent forms of acute leukemia in adults, is a malignant clonal disorder originating from hematopoietic stem cells (1). Characteristic features of AML include uncontrolled proliferation of leukemia stem cells (LSCs), impaired generation of functionally mature hematopoietic cells, bone marrow infiltration, and epigenetic dysregulation (2). The resultant suppression of normal erythrocyte, leukocyte, and platelet production leads to hallmark clinical symptoms such as hemorrhage, fever, and bone pain (3). In 2020, AML accounted for 2.5% of new cancer diagnoses and 3.1% of cancer-related deaths globally, ranking among the leading causes of cancer mortality (4). Prognosis remains poor, with some patients developing central nervous system involvement, further exacerbating disease severity and life-threatening complications (5). Standard therapy involves intensive induction chemotherapy followed by consolidation or allogeneic stem cell transplantation. Despite high initial response rates, relapse is common, and the 5-year survival rate remains at only 29.3% (6). Consequently, molecularly targeted therapies are garnering increasing attention. Small molecule inhibitors targeting key proteins and signaling pathways—such as FLT3, IDH, and BCL2—have shown clinical promise but often yield suboptimal and transient responses, contributing to therapeutic failure (7–9). Elucidating the molecular mechanisms underlying AML pathogenesis is therefore critical for advancing more effective treatment strategies.

Autophagy, a cellular degradation pathway, facilitates the removal of dysfunctional, damaged, or senescent cells, organelles, and proteins *via* lysosomal transport. Since the elucidation of its mechanism in 2016 (10), various autophagy subtypes—macroautophagy, chaperone-mediated autophagy, microautophagy, and selective autophagy—have garnered substantial research interest. Mitophagy, a mitochondria-specific form of autophagy, plays a pivotal role in maintaining mitochondrial quality control and cellular homeostasis while mitigating oxidative stress (11, 12). Hypoxia-induced mitophagy, mediated by receptors such as BNIP3, NIX, and FUNDC1, is notably upregulated under low-oxygen conditions (13). Both mitochondrial respiration and mitophagy are critically involved in the maintenance of LSCs (14–16). Experimental evidence indicates that disruption of mitochondrial homeostasis heightens the sensitivity of functional LSCs and hypoxic AML progenitors to autophagy inhibition (17). Moreover, elevated expression of the mitophagy receptor optineurin (OPTN) has been shown to suppress AML cell proliferation (18).

This study aims to investigate the role of mitophagy-related genes (MRGs) in the pathogenesis and progression of AML, offering valuable insights into early diagnosis and prognosis. A set of mitophagy-associated biomarkers was identified through integrated bioinformatics approaches, including differential gene expression analysis and machine learning, followed by the construction of a risk prediction model. Functional enrichment analysis, immune infiltration profiling, tumor mutational burden (TMB) assessment, and drug sensitivity evaluation were further conducted to elucidate the biological relevance and therapeutic potential of these biomarkers. The findings provide a foundation for the development of more stable, efficacious, and safer therapeutic targets, offering novel clinical guidance for AML treatment.

## 2 Materials and methods

### 2.1 Data source

The AML-related dataset TCGA-LAML, comprising gene expression matrices and clinical data for 132 patients, was retrieved from the University of California, Santa Cruz (UCSC) Xena platform (https://xenabrowser.net/datapages/). Additionally, two gene expression profiles, GSE24395 (platform GPL610) and GSE146173 (platform GPL18460), were obtained from the Gene Expression Omnibus (GEO) database (https://www.ncbi.nlm.nih.gov/gds). The GSE24395 dataset contained 12 control and 5 AML patient samples, which were sequenced on the GPL6106 platform. Transcriptome raw counts were downloaded, and the mapping relationship between probe IDs and gene symbols was extracted according to the annotation file of the sequencing platform. The probe with the highest mean expression value for each gene symbol was retained, and data were processed using a 75th percentile shift normalization algorithm. The GSE146173 dataset included 202 AML patient samples, which were sequenced on the GPL18460 platform. After downloading the raw counts, ENSEMBL IDs were converted to gene symbols using the org.Hs.eg.db package (v 3.16.0) (19). Gene lengths were then obtained from the GPL18460 annotation file to calculate reads per kilobase (RPK). Further, fragments per kilobase of transcript per million mapped reads (FPKM) was computed, and log_2_ transformation (log_2_^(FPKM + 1)^) was applied to the FPKM values to stabilize variance and facilitate subsequent analyses. Based on the October 16, 2023 version of the Kyoto Encyclopedia of Genes and Genomes (KEGG) human gene set (kegg_hsa.gmt), genes explicitly annotated to the “hsa04137_Mitophagy” pathway were screened. After removing duplicates, 72 mitophagy-related genes (MRGs) were obtained (**Supplementary Table S1**).

### 2.2 Consensus clustering analysis and differential expression analysis

To identify prognostically relevant MRGs, their expression profiles were extracted from the TCGA-LAML dataset and subjected to univariate Cox regression analysis (*P* < 0.05). Genes meeting this threshold were designated as prognostic MRGs. Using these genes, consensus clustering was performed on TCGA-LAML samples with the ConsensusClusterPlus package (v1.62.0) (20) to stratify molecular subtypes. The optimal number of clusters was determined based on the cumulative distribution function (CDF) curves and the delta area index. To assess survival differences among these subtypes, Kaplan-Meier (K-M) survival curves were constructed based on overall survival (OS) using the TCGA-LAML cohort. To further refine the analysis, differentially expressed genes (DEGs) were identified in two comparisons: (1) between patients with AML and healthy controls in GSE24395 (denoted DEGs1), and (2) between the identified molecular subtypes within TCGA-LAML (denoted DEGs2). The limma (v1.38.0) and DESeq2 (v1.38.0) packages were applied for DEG analysis (21, 22), with thresholds of |log_2_FC| > 1.5 and adjusted *P*-value < 0.05. The top 10 upregulated and downregulated genes from each comparison were visualized using volcano plots and heatmaps generated with the ggplot2 package (v3.4.1) (23).

### 2.3 Acquisition and functional enrichment analysis of candidate genes

Candidate genes were identified by intersecting DEGs1 and DEGs2. Functional interpretation of these candidates was conducted through Gene Ontology (GO) and KEGG pathway enrichment analyses using the clusterProfiler package (v4.7.1.3) (24), applying a significance cutoff of *P* < 0.05. Additionally, protein-protein interaction (PPI) networks for the candidate genes were constructed using the STRING database, with a minimum interaction confidence score set at 0.15.

### 2.4 Establishment and verification of risk model

The expression profiles of candidate genes in the TCGA-LAML dataset were first analyzed through univariate Cox regression (*P* < 0.05) to identify genes with prognostic significance. Subsequently, the least absolute shrinkage and selection operator (LASSO) regression was performed using the glmnet package (v4.1.4) to further refine the biomarker selection for constructing a prognostic risk model in the TCGA-LAML dataset (25).

A risk score was then computed for each patient with AML in the TCGA-LAML dataset based on the expression levels of selected biomarkers. Patients were stratified into high- and low-risk groups using the median risk score as a cutoff. Risk score distribution plots were generated to illustrate the separation between these two groups. K-M survival curves were constructed using the survminer package (v0.4.9) to evaluate differences in OS between the high- and low-risk cohorts (26). The predictive performance of the risk model was further assessed by time-dependent receiver operating characteristic (ROC) curves, plotted using the survivalROC package (27). In addition, the risk model was validated using risk scores, KM survival analysis, and ROC curve evaluation in the GSE146173 dataset. What’s more, to further validate the reliability of a risk model as a clinical predictive tool for AML patients.

### 2.5 Independent prognostic analysis and nomogram construction

To assess the clinical utility of the model, TCGA-LAML samples were further categorized based on clinical variables including age (≤ 60/>60), gender (male/female), race (Asian/Black or African American/White), cytogenetic risk category (favorable/intermediate or normal/poor), treatment history (yes/no), and French-American-British (FAB) classification (M0–M7). Stratified K-M survival analyses were conducted for each subgroup using the survival package (v 3.5.3) (28). To identify independent prognostic indicators, both risk scores and clinical features were subjected to univariate Cox regression analysis. Variables meeting the significance threshold (*P* < 0.05) were subsequently tested for proportional hazards (PH) assumptions (*P* > 0.05) before inclusion in multivariate Cox regression, through which independent predictors of survival were identified.

A prognostic nomogram was then developed using the rms package based on the independently validated prognostic factors, allowing for the prediction of 1-, 3-, and 5-year OS in patients with AML (26). The predictive accuracy and calibration of the nomogram were validated using calibration plots generated with the rms package (v6.7.1) and time-dependent ROC curves plotted *via* the timeROC package (26, 29).

Finally, to investigate the relationship between risk stratification and clinical features, clinical annotations from TCGA-LAML were analyzed. Chi-square tests were used to assess statistical differences in clinical characteristics and survival outcomes between high- and low-risk groups (30).

### 2.6 Gene set enrichment analysis of different risk groups

In this study, differential gene expression analysis between high- and low-risk groups was conducted using the DESeq2 package (v1.38.0) (22). Genes were ranked by log_2_FC, sorted in descending order to prioritize those with the most significant expression differences. To elucidate the biological processes associated with these differences, GSEA was performed using the clusterProfiler package (v4.7.1.3) (24), referencing the GO Biological Process (GO-BP) category from the MSigDB database (c5.go.bp.v2023.1.Hs.symbols.gmt; https://www.gsea-msigdb.org/). Pathways meeting the criteria |normalized enrichment score (NES)| > 1 and *P* < 0.05 were considered significant, and the top five enriched pathways were selected for visualization.

### 2.7 Immune infiltration analysis

To characterize the immune landscape within the AML samples from the TCGA-LAML dataset, the CIBERSORT algorithm was applied to estimate the relative abundance of 22 immune cell types (31). Differences in immune cell infiltration between risk groups were evaluated using the Wilcoxon signed-rank test (*P* < 0.05). Additionally, Spearman correlation analysis was employed to explore associations between immune cell proportions and risk scores, with results considered significant when |r| > 0.4 and *P* < 0.05. Immune checkpoint gene expression analysis was also performed by extracting the expression levels of eight key immune regulatory genes—CD274, LAG3, TGFB1, TNFSF13, CD4, CD40, CD80, and CD276—from the TCGA-LAML dataset. Group-wise comparisons of these genes were conducted using the Wilcoxon test (*P* < 0.05), and their correlation with risk scores was visualized *via* scatter plots. To further elucidate the interaction between key biomarkers and the tumor immune microenvironment, relevant immune cell reporter genes and immune regulatory genes were retrieved from the TISIDB database (http://cis.hku.hk/TISIDB/). Spearman correlation analysis was then conducted between selected biomarkers and these immune-related genes, with |r| > 0.4 and *P* < 0.05 indicating statistically significant associations.

### 2.8 Drug sensitivity analysis

To explore the association between biomarkers and chemotherapeutic response, half-maximal inhibitory concentration (IC_50_) values for 138 commonly used chemotherapeutic agents were estimated using the pRRophetic package (v0.5) (32) across all TCGA-LAML samples, followed by comparative analysis between risk groups. In addition, the corrplot package (v 0.92) (33) was used to evaluate the correlations between the top 20 drugs with significant differences in IC50 between the high-risk and low-risk groups and the 5 biomarkers through Spearman correlation analysis (|r| > 0.4 and *P* < 0.05).

### 2.9 Molecular regulatory network construction

To elucidate potential regulatory mechanisms of the identified biomarkers in AML, upstream elements were predicted. miRNAs targeting the biomarkers were identified *via* miRDB (https://mirdb.org/) and miRanda (http://www.microrna.org/microrna/home.do). miRNAs common to both databases were then used to predict interacting long non-coding RNAs (lncRNAs) through the starBase platform (http://starbase.sysu.edu.cn/). Transcription factors (TFs) potentially regulating the biomarkers were inferred from the ChEA3 database (https://maayanlab.cloud/chea3). The lncRNA–miRNA–mRNA and mRNA–TF regulatory networks were visualized using Cytoscape (v3.10.2).

### 2.10 Validation of biomarker expression

Expression levels of the biomarkers were examined in the GSE24395 dataset, with statistical significance defined at *P* < 0.05. Validation of biomarker expression was further conducted *via* quantitative reverse transcription PCR (qRT-PCR). Whole blood samples from fifteen patients with AML and fifteen healthy donors, collected at the Second Hospital of Hebei Medical University, were used for qRT-PCR analysis. All participants provided written informed consent, and the study was approved by the institutional ethics committee.

Total RNA was extracted from frozen samples using TRIzol reagent (Ambion, China). Equal amounts of mRNA were reverse-transcribed to synthesize cDNA. qPCR was conducted using the 2 × Universal Blue SYBR Green qPCR Master Mix kit (Servicebio, China), following standard protocols. Gene expression levels were quantified using the 2^–ΔΔCT^ method. Primer sequences are listed in **Supplementary Table S2**.

### 2.11 Statistical analysis

All bioinformatics analyses were performed using R software (v4.2.2), with *P* < 0.05 considered statistically significant.

## 3 Results

### 3.1 Consensus clustering analysis among patients with AML

**Figure 1:**
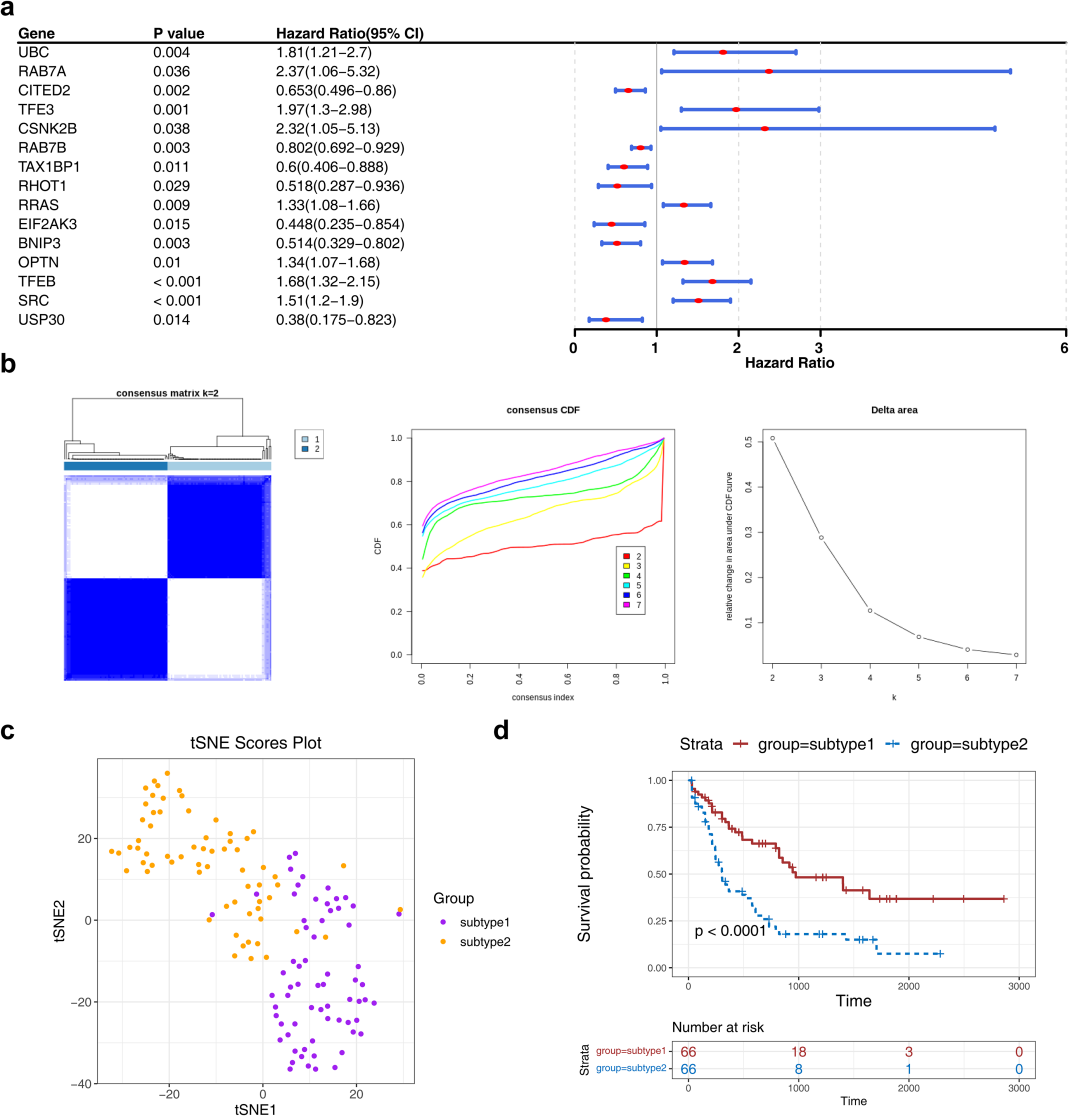
The consensus clustering analysis among AML patients. **(a)** The forest plot of MRG associated with prognosis in AML patients. The figure showed the names of MRGs, P-values, HR values, and 95% CIs. An HR value greater than 1 was indicated to be a risk factor for AML, while an HR value less than 1 was indicated to be a protective factor. **(b, c)** The consensus cluster analysis of prognosis-related MRGs in AML patients.AML patients were classified into two subtypes based on MRGs. **(d)** The K-M curve of different subtypes. Red represents subtype 2, and blue represents subtype 1.

Expression profiling of 72 MRGs in the TCGA-LAML dataset identified 15 prognostic MRGs through univariate Cox regression analysis, which were subsequently used for consensus clustering (**Figure 1a**). Based on the CDF curves and delta area index, the optimal number of clusters was determined as k=2, and the samples in the TCGA-LAML dataset were divided into two subtypes, namely subtype 1 and subtype 2 (**Figure 1b**)—with their distribution visualized *via* t-SNE dimensionality reduction (**Figure 1c**). K-M survival analysis revealed a statistically significant difference in OS between the subtypes, with subtype 1 associated with a poorer prognosis (**Figure 1d**).

### 3.2 A total of 26 genes were identified as candidate genes

**Figure 2:**
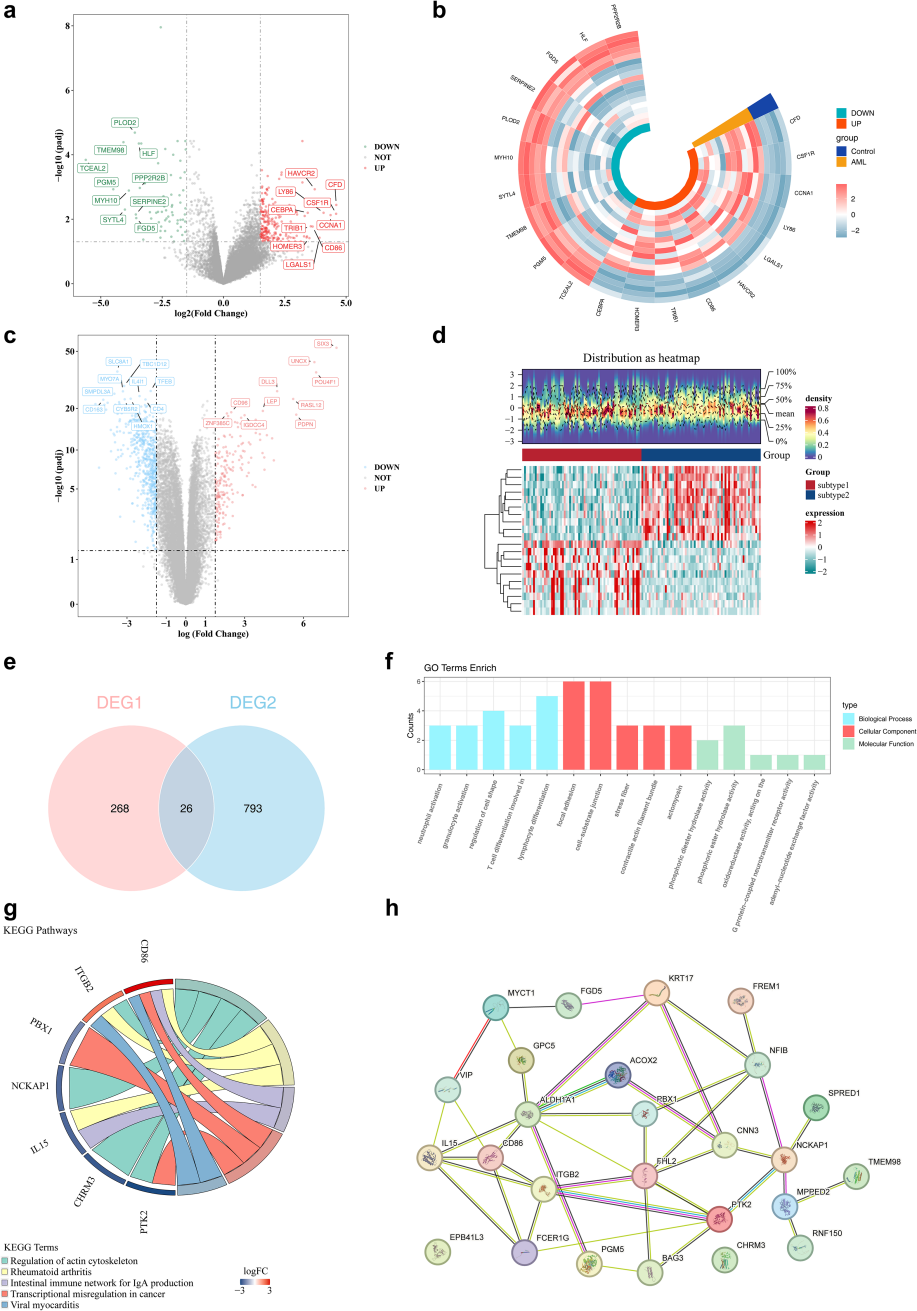
The identification of differential genes among subtypes. **(a, b)** The volcano and heat maps of differential genes in the training set. In the volcano plot, red dots and green dots represent up - regulated and down-regulated genes respectively, while gray dots represent genes with no significant difference. In the heatmap, red and green represent up - regulated and down-regulated genes respectively. **(c, d)** The volcano and heat maps of differential genes between subtype 1/2. **(e)** The venn diagram of DEGs1 genes and DEGs2 genes. **(f)** The results of GO enrichment analysis of differential genes. **(g)** The results of KEGG enrichment analysis of differential genes. **(h)** The construction of PPI networks for candidate genes. Nodes represent proteins, and lines represent interactions.

Differential expression analysis was conducted to identify disease- and subtype-specific transcriptomic alterations. A total of 294 DEGs1, including 223 upregulated and 71 downregulated genes, were identified between AML samples and controls in the GSE24395 dataset (**Figures 2a, b**). In parallel, a total of 819 DEGs2 (205 upregulated and 614 downregulated) were identified between subtype 1 and subtype 2 in the TCGA-LAML dataset (**Figures 2c, d**). The intersection of DEGs1 and DEGs2 yielded 26 candidate genes for further analysis (**Figure 2e**, **Supplementary Table S3**). These 26 genes were subjected to functional enrichment analysis. GO analysis revealed enrichment in 348 terms, comprising 277 biological processes (BPs), 36 cellular components (CCs), and 35 molecular functions (MFs). The top five enriched terms were predominantly related to neutrophil activation, focal adhesion, and phosphoric diester hydrolase activity, among others (**Figure 2f**). KEGG pathway analysis identified 15 significantly enriched signaling pathways, with the top five including regulation of the actin cytoskeleton and rheumatoid arthritis (**Figure 2g**). Furthermore, PPI network analysis highlighted 24 interacting genes, forming key interaction axes such as *PTK2*–*ITGB2*, *PTK2*–*BAG3*, and *BAG3*–*FHL2* (**Figure 2h**).

### 3.3 *ITGB2*, *VIP*, *PTK2*, *FHL2*, and *BAG3* were identified as biomarkers for constructing risk models

**Figure 3:**
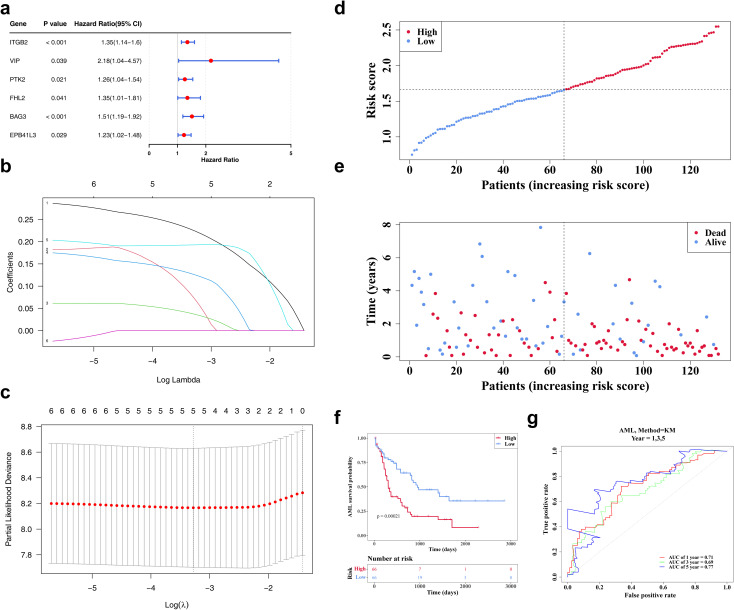
The identification of biomarkers. **(a)** The forest map of prognosis-related candidate genes. It shows the P-values, HR, and 95% CIs for multiple genes, which are used to evaluate the impact of each gene on AML risk. **(b, c)** The LASSO regression coefficient graph and cross-validation graph based on penalty term. **(d)** The risk curves of the TCGA-LAML sample. The red line represents the high-risk group, and the blue line represents the low-risk group. **(e)** The survival status distributions for the TCGA-LAML sample. Red dots represent deceased patients, while blue dots represent surviving patients. **(f)** The TCGA-AML K-M curves for high and low risk groups. Red represents the high-risk group, and blue represents the low-risk group. **(g)** The ROC curve of the model in TCGA-AML. An AUC value greater than 0.6 indicates that the model has certain predictive performance.

Lasso regression analysis was conducted on six genes (*ITGB2*, *VIP*, *PTK2*, *FHL2*, *BAG3*, *EPB41L3*) previously identified *via* univariate Cox regression (**Figure 3a**). The analysis retained five genes—*ITGB2*, *VIP*, *PTK2*, *FHL2*, and *BAG3*—with non-zero regression coefficients, which were subsequently selected as biomarkers for constructing the prognostic risk model (**Figures 3b, c**). The risk score was calculated using the following formula: Risk score = 0.22405735 × *ITGB2* expression + 0.07079774 × *VIP* expression + 0.03667777 × *PTK2* expression + 0.12551123 × *FHL2* expression + 0.19284549 × *BAG3* expression. Based on the median risk score, patients with AML were stratified into high- and low-risk groups (n = 66 per group). A clear positive association between risk score and mortality was observed (**Figures 3d, e**). K-M survival analysis demonstrated significantly reduced OS in the high-risk group (**Figure 3f**). ROC curve analysis revealed areas under the curve (AUCs) of 0.71, 0.69, and 0.77 for 1-, 3-, and 5-year survival, respectively, indicating robust predictive performance of the model (**Figure 3g**).

The model’s predictive validity was externally verified using the GSE146173 dataset. Similar trends were observed, with high-risk patients exhibiting reduced survival (**Supplementary Figures S1a–c**). AUC values consistently exceeded 0.6, aligning well with results from the TCGA-LAML cohort (**Supplementary Figure S1d**), confirming the model’s reliability and generalizability as a clinical prognostic tool for AML.

### 3.4 The risk score, age, and treatment were independent prognostic factors for AML

**Figure 4:**
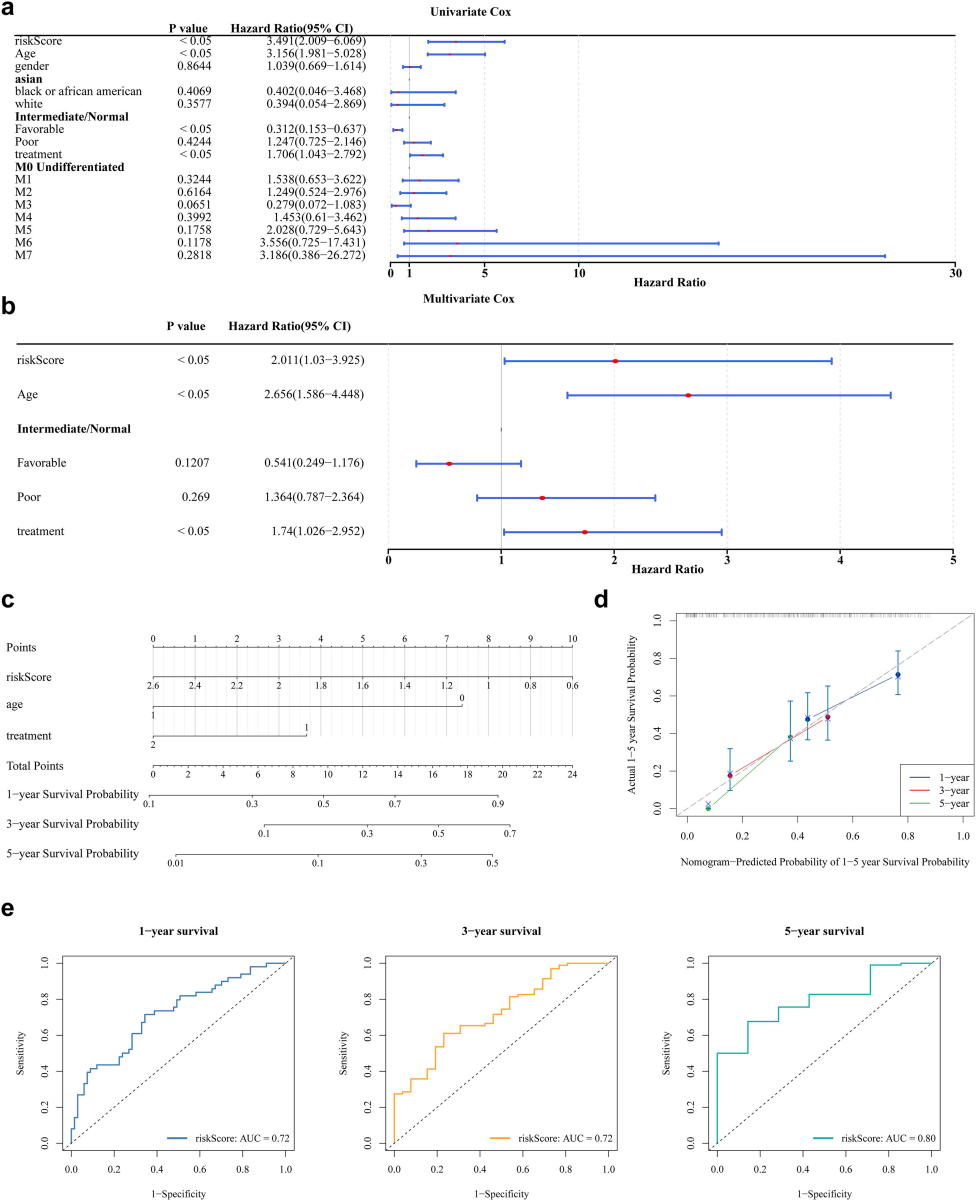
Nomogram model construction and evaluation. **(a)** The results of univariate Cox analysis. It shows the P-values, HR values, and 95% CI values for different variables. **(b)** The results of multivariate Cox regression analysis. **(c)** The construction of nomogram. In the variable “age”, values greater than 65 years are coded as 1, and those less than 65 years are coded as 0. In the variable “treatment”, patients who received treatment are coded as 2, while those who did not receive treatment are coded as 1. In the “risk score”, the values ranging from 2.6 to 0.6 correspond to the risk values of the samples. Each variable is assigned a score, and the total score is calculated based on the scores of all variables to predict the 1-year, 3-year, and 5-year survival probabilities of AML. **(d)** The calibration curve showed that the accuracy of the nomogram was relatively high and validated the model performance of AML. **(e)** The ROC curve indicates that the nomogram model has an excellent predictive value for AML (AUC>0.6).

Analysis of clinical parameters revealed that age, health status, treatment regimen, and FAB classification significantly influenced the outcomes of patients with AML. In particular, patients aged ≤60 years, with favorable health, no prior treatment, or FAB classification M3, showed markedly improved survival (**Supplementary Figures S2a–f**). Univariate Cox regression analysis confirmed the prognostic significance of risk score, age, health status, and treatment, with consistency across PH assumption testing (**Figure 4a**). Multivariate Cox regression further identified risk score, age, and treatment as independent prognostic factors (**Figure 4b**). A prognostic nomogram incorporating these independent variables was developed to estimate individualized survival probabilities for patients with AML (**Figure 4c**). The calibration curve demonstrated strong concordance between predicted and observed survival outcomes (**Figure 4d**). ROC curve analysis of the nomogram yielded AUC values exceeding 0.6 at 1, 3, and 5 years, supporting its high predictive accuracy (**Figure 4e**).

Comparative analysis of clinical characteristics between high- and low-risk groups indicated significant differences in age, health status, FAB classification, and OS (**Supplementary Figure S3a**). Additionally, GSEA identified 731 significantly enriched functional terms between risk groups, with the top five—including mononuclear cell migration and cellular response to biotic stimulus—highlighted in the visualization (**Supplementary Figure S3b**).

### 3.5 Seven immune cells differed significantly by risk group

**Figure 5:**
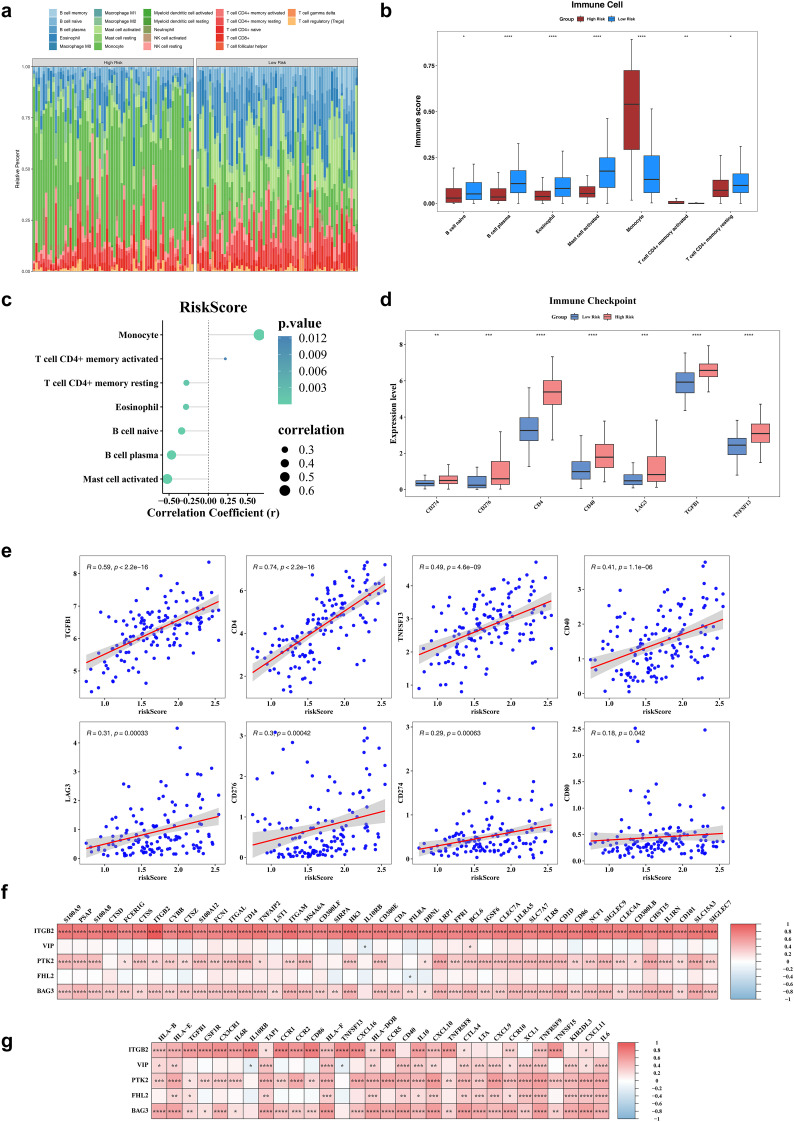
Immune infiltration analysis. **(a)** The heatmap of immune infiltrating cell profiles in high and low risk groups. **(b)** The differences in immune infiltrating cell abundance between diseased healthy groups. **(c)** The correlation analysis of risk scores and differential immune infiltrating cells. **(d)** The significantly different immune checkpoints in high and low risk groups. **(e)** The immunization checkpoints correlate with risk scores. **(f)** The heatmap of risk model genes correlating with cell report genes. **(g)** The heatmap of risk model genes correlating with Immune regulators genes. *P < 0.05, **P< 0.01, ***P < 0.001, ****P < 0.0001.

Immune cell infiltration was assessed using the CIBERSORT algorithm, which estimated the relative abundance of 22 immune cell types (**Figure 5a**). Significant differences in immune infiltration were observed between risk groups for seven cell types. Specifically, five immune cells—naive B cells, plasma B cells, eosinophils, activated mast cells, and memory-resting CD4^+^ T cells—exhibited significantly reduced infiltration in the high-risk group. In contrast, monocytes and memory-activated CD4^+^ T cells showed no significant decline (**Figure 5b**). Correlation analysis revealed a strong positive association between monocyte infiltration and risk scores (r > 0.4, *P* < 0.05), while plasma B cells and activated mast cells were negatively correlated with risk scores (r < -0.4, *P* < 0.05) (**Figure 5c**). Among eight examined immune checkpoints, seven (CD274, LAG3, TGFB1, TNFSF13, CD4, CD40, and CD276) were significantly overexpressed in the high-risk group, with CD80 as the only exception (**Figure 5d**). Risk scores exhibited positive correlations with the expression levels of all eight immune checkpoints (**Figure 5e**). Additionally, a total of 315 cell reporter genes and 65 immune regulatory genes showed significant associations with the biomarkers, as depicted in heatmaps (**Figures 5f, g**).

### 3.6 A total of 84 drugs were sensitive to AML

Drug sensitivity analysis identified 84 compounds with significantly different IC50 values between the two risk groups. Notable examples included AS601245, BIBW2992, and Bleomycin (**Supplementary Figures S4a, b**), suggesting distinct therapeutic vulnerabilities associated with molecular risk stratification. Furthermore, AS601245, BIBW2992, and Bleomycin all showed strong negative correlations with the 5 biomarkers (r < -0.3) (**Supplementary Figure S4c**).

### 3.7 Various regulatory elements of *ITGB2, VIP, PTK2, FHL2* and *BAG3*

**Figure 6:**
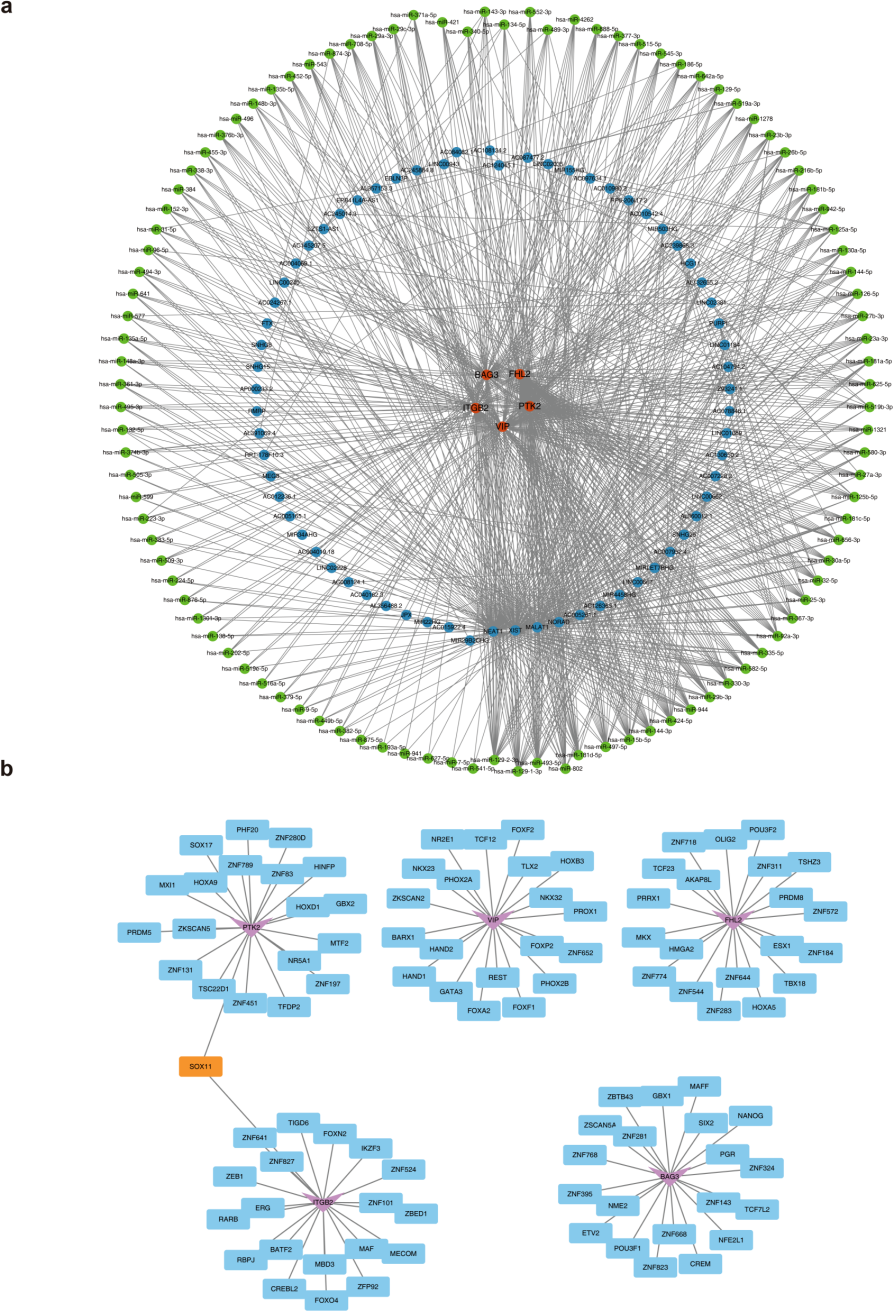
The molecular regulatory network of biomarkers. **(a)** The lncRNA-miRNA-mRNA regulatory network. Orange represents biomarkers, blue represents lncRNAs, and green represents miRNAs. **(b)** The TFs-mRNA regulatory network. The pink represents biomarkers, blue represents TFs, and orange represents shared TFs.

Regulatory elements of *ITGB2*, *VIP*, *PTK2*, *FHL2*, and *BAG3* were systematically predicted. The constructed lncRNA–miRNA–mRNA regulatory network included miRNAs such as hsa-miR-135b-5p, hsa-miR-452-5p, and hsa-miR-543, which target the biomarkers, and upstream lncRNAs including FTX, HCG11, and PURPL that regulate these miRNAs (**Figure 6a**). TFs were also predicted: SOX11 targeting *ITGB2* and *PTK2*, TLX2 regulating *VIP*, AKAP8L acting on *FHL2*, and SIX2 targeting *BAG3* (**Figure 6b**). These regulatory elements are critical for elucidating the mechanistic roles of MRGs in AML progression.

### 3.8 The expression of biomarkers

**Figure 7:**
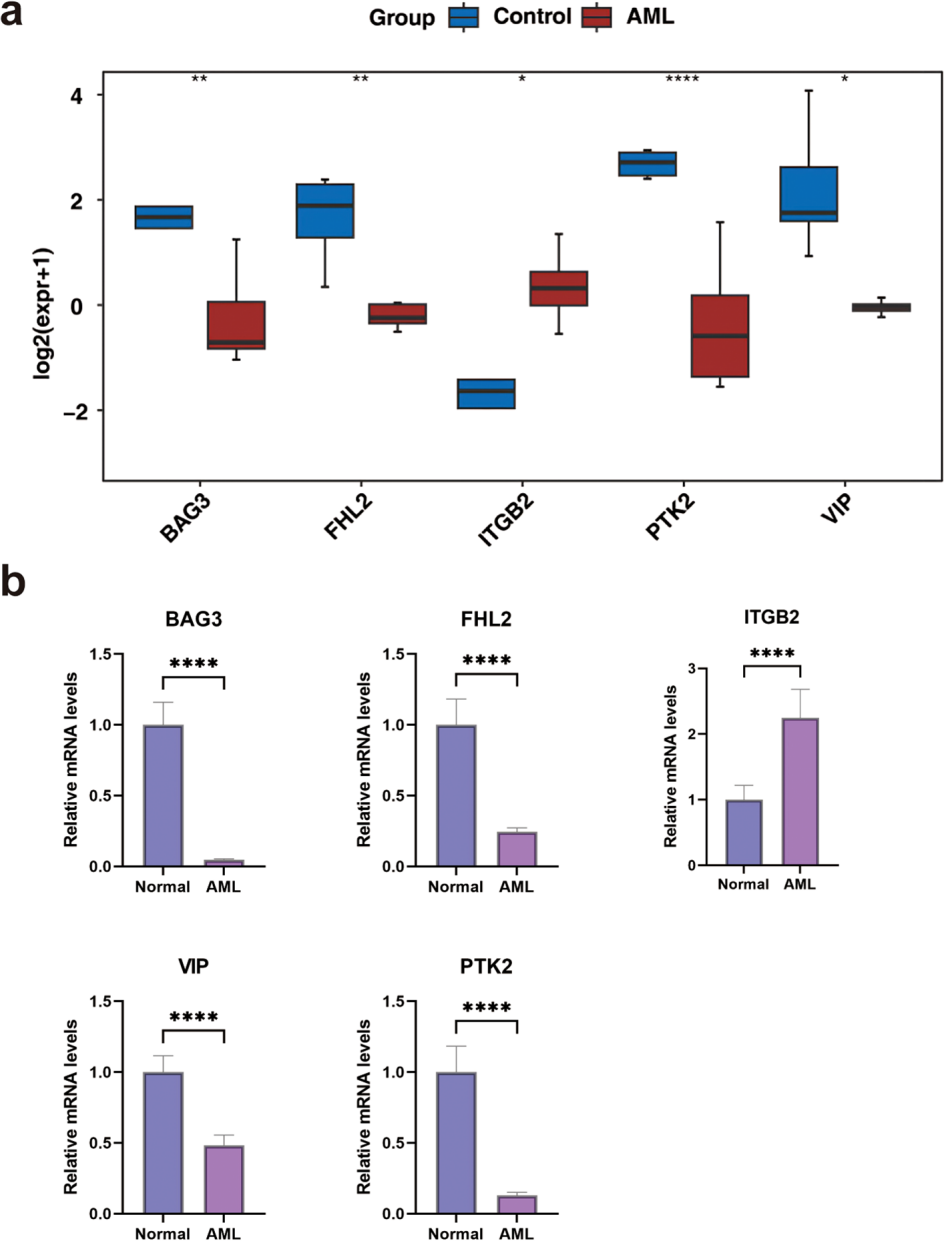
The expression levels of biomarkers. **(a)** In the GSE24395 dataset, the expression levels of biomarkers between AML and control groups. **(b)** The qRT-PCR of biomarkers. *P < 0.05, **P< 0.01, ***P < 0.001, ****P < 0.0001.

Expression validation using the GSE24395 dataset indicated significant downregulation of *VIP*, *PTK2*, *FHL2*, and *BAG3* in AML samples, while ITGB2 was upregulated (**Figure 7a**). These findings were further corroborated by qRT-PCR, which confirmed reduced expression of *PTK2*, *FHL2*, *VIP*, and *BAG3*, and elevated expression of *ITGB2* in AML samples (**Figure 7b**).

## 4 Discussion

AML, a group of highly heterogeneous hematologic malignancies, is characterized by poor therapeutic outcomes and short survival durations, particularly among patients with intermediate- to high-risk disease or refractory/relapsed forms. This clinical challenge underscores an urgent need to expand therapeutic strategies for AML. Although the role of mitophagy and mitochondrial homeostasis in cancer has been increasingly recognized (34–36), including the established function of SQSTM1 in leukemia progression (14), the specific mechanisms by which MRGs drive AML heterogeneity remain incompletely defined.

In this study, we conducted univariate Cox regression analysis to screen 15 prognostically related MRGs for concordance cluster analysis. Ultimately, AML patients were divided into two distinct subtypes, namely subtype 1 and subtype 2. Cluster analysis based on MRGs uncovered significant molecular heterogeneity within the AML patient population (37). The marked survival differences between the subtypes imply potential discrepancies in sensitivity to existing treatments. This suggests that clinicians could personalize treatment strategies according to the subtype - specific characteristics (38). The lower survival rates observed in subtype 1 patients indicate that this subtype might be associated with a more active aberrant mitophagy process or accompanied by other molecular events linked to poor prognosis. These abnormalities may contribute to the unfavorable prognosis of subtype 1 patients by potentially accelerating leukemia cell proliferation, inducing drug resistance, or facilitating immune surveillance evasion (18, 39). Furthermore, subtype classification can aid in patient stratification during clinical trials, thereby enhancing the accuracy and effectiveness of novel therapy evaluation (40).

In this study, 26 candidate genes were obtained by intersection analysis of 294 DEGs1 from between AML and control groups and 819 DEGs2 from between different subtypes. Subsequent univariate Cox and Lasso regression analyses identified five key biomarkers—*ITGB2*, *VIP*, *PTK2*, *FHL2*, and *BAG3*—for constructing a prognostic risk model. Among them, *ITGB2*, encoding the CD18 integrin subunit, was initially identified in leukocytes, where it mediates adhesion to endothelial cells and facilitates transendothelial migration. Prior research has implicated *ITGB2* in drug resistance mechanisms to mitoxantrone and idarubicin in AML (41, 42). Notably, *ITGB2* is significantly overexpressed in AML and positively correlates with poor prognosis, potentially *via* interaction with STK10 (43). Collectively, these findings position *ITGB2* as a clinically relevant biomarker for AML diagnosis and prognosis. Mechanistically, ITGB2 may influence mitophagy through its role in cell adhesion and stress response signaling. Studies suggest that integrin-mediated adhesion pathways can modulate mitochondrial homeostasis under cellular stress, potentially linking ITGB2 to dysregulated mitophagy in AML progression. *VIP* (vasoactive intestinal peptide), a 28-amino acid neuropeptide initially isolated from porcine duodenum, is widely distributed across multiple physiological systems including the nervous, reproductive, and respiratory systems (44). Although *VIP* has been shown to mitigate bleomycin-induced pulmonary fibrosis by restoring autophagy in alveolar epithelial cells (45), its role in hematologic malignancies remains unexplored. Notably, VIP may regulate mitochondrial function via the cAMP/PKA pathway, which is implicated in coordinating energy metabolism and mitophagy. This pathway could contribute to mitochondrial quality control in leukemia cells, offering a potential mechanistic link between VIP and mitophagy in AML (46–48). *PTK2* (protein tyrosine kinase 2), also known as focal adhesion kinase (FAK), functions as a non-receptor tyrosine kinase regulating integrin and growth factor receptor signaling (49). Elevated expression of *PTK2* has been documented in various malignancies such as lung cancer, hepatocellular carcinoma, and lymphocytic leukemia (50, 51). Furthermore, *PTK2* has been identified as an independent prognostic factor in AML, particularly in intermediate-risk cohorts. PTK2 may further influence mitophagy through its modulation of mitochondrial membrane potential and ROS production. Crosstalk between focal adhesion kinase signaling and autophagy pathways could disrupt mitochondrial quality control, promoting survival of leukemic stem cells (52–54). It also serves as a prognostic discriminator among patients harboring unfavorable FLT3/NPM1 mutations with concurrent low PTK2B or LYN expression (55). The FHL2 protein is localized in adhesion plaques, the cytoplasm, and the nucleus, where it interacts with a broad array of TFs and tumor-associated proteins (56, 57). Its expression is markedly elevated in gastric cancer tissues compared to chronic gastritis, with overexpression closely linked to gastric tumorigenesis (58). Emerging evidence also implicates *FHL2* in leukemogenesis (59). Beyond its oncogenic potential, *FHL2* plays a pivotal role in regulating the survival of hematopoietic stem cells under both homeostatic and stress conditions. While not directly linked to mitophagy, this role in mitochondrial dynamics provides a novel angle to explore its contribution to mitophagic flux in AML (60). *BAG3* (Bcl-2-associated athanogene 3), a member of the BAG protein family, exerts its biological functions by interacting with target proteins *via* multiple structural motifs, including the BAG domain, WW domain, and PXXP motif. It is involved in a range of cellular processes such as autophagy, apoptosis, embryogenesis, cytoskeletal reorganization, and cell migration (61, 62). BAG3 is aberrantly expressed in several solid tumors and germ cell leukemias (62, 63), and has been proposed as a potential therapeutic target in cancers including glioma, pancreatic cancer, and ovarian carcinoma. Additionally, BAG3 is upregulated in response to various cellular stressors, facilitating selective autophagy to maintain intracellular homeostasis (64–68). As a co-chaperone, BAG3 directly interacts with HSP70 via its BAG domain to facilitate selective autophagy of damaged organelles, including mitochondria (69, 70). This HSP70-BAG3 complex recruits LC3 to ubiquitinated substrates, positioning BAG3 as a critical regulator of mitophagic clearance in cancer cells (71). Its overexpression may thus confer a survival advantage to AML blasts by enhancing mitochondrial turnover. In summary, five biomarkers—*ITGB2*, *VIP*, *PTK2*, *FHL2*, and *BAG3*—were identified in this study as being associated with AML. These genes appear to be functionally relevant to AML pathogenesis and may serve as valuable molecular targets for future diagnostic and therapeutic strategies.

The results of this study showed that in the TCGA-LAML data set, the risk score was significantly positively correlated with patient mortality. The difference in survival between high- and low-risk groups was statistically significant, and the 5-year ROC curve AUC value was 0.77. These results are highly consistent with the range of performance of AML prognostic models reported in previous studies (72). Furthermore, the consistency between the results and the external validation set (GSE146173) fully reflects the reliability and universality of the model in predicting long-term prognosis. Additionally, from the association analysis of clinical characteristics and prognosis, patients aged ≤60 years, in good health, untreated, or FAB-typed as M3 had a significantly higher survival rate. This finding is consistent with the clinical perception that younger patients are in better physical condition and that M3 AML patients are sensitive to targeted therapies (73). The nomogram model integrates independent prognostic factors such as risk score, age, and treatment type. Its predictive accuracy was verified by calibration curve and ROC analysis, providing a visual decision-making tool for clinical practice (74). For example, in elderly patients with a high-risk score and comorbidities, the model may suggest a poor prognosis and the need to prioritize intensive supportive care or novel therapies (75). Younger patients with a lower risk score may benefit more from standard care (76). GSEA enrichment results in high- and low-risk groups (monocyte migration, biostimulatory response pathways) revealed that the risk model may be associated with immune microenvironment heterogeneity or abnormal regulation of inflammation in AML (77). The activation of monocyte migration-related pathways may be associated with leukemia cell infiltration or immune escape mechanisms. This provides directions for further exploration of AML pathogenesis and the development of therapeutic strategies targeting immune checkpoints or inflammatory signaling pathways (78).

Immunotherapy based on immune checkpoint inhibitors (ICIs) has garnered increasing attention for its efficacy in treating advanced, metastatic, and recurrent malignancies. By disrupting inhibitory checkpoint signaling, ICIs restore anti-tumor immune responses. Clinical success has been demonstrated in cancers such as melanoma, lung, and prostate cancer (79–82). APRIL/TNFSF13, an immune checkpoint molecule, is overexpressed in colorectal and glioma tissues and has been linked to adverse pathological features and poor prognosis, suggesting its potential as a therapeutic target (83, 84). In addition, the therapeutic relevance of tumor-infiltrating B cells has been established in colorectal cancer (85).

Although immune checkpoint and immune infiltration analyses have significantly contributed to predictive modeling and drug sensitivity profiling in solid tumors, similar investigations in AML remain limited. The present analysis highlights monocytes, plasma B cells, and activated mast cells as key immune populations associated with risk stratification in AML. Moreover, targeting immune checkpoints such as TGFB1, TNFSF13, CD4, and CD40 may enhance immunotherapeutic efficacy in AML. Nonetheless, further mechanistic studies are required to elucidate the specific roles of these immune cells and immune checkpoints within the AML microenvironment.

Accumulating evidence highlights the pivotal role of mitophagy in the pathogenesis of AML, with its modulation offering potential to enhance therapeutic efficacy and overcome drug resistance. Elucidating specific molecular mechanisms and identifying precise regulatory targets thus represent promising strategies for advancing AML treatment. In the present study, MRGs were associated with AML through differential expression analysis, consensus clustering, univariate Cox regression, LASSO modeling, and related computational approaches. Five prognostic biomarkers were identified and incorporated into a risk prediction model, providing a theoretical framework for further exploration of MRGs in AML. Nevertheless, the clinical utility of these biomarkers remains constrained by limited sample size, and additional mechanistic studies and larger cohorts are essential to validate their translational potential in AML therapy.

Although this study used bioinformatics to screen five mitophagy genes linked to AML prognosis and built a risk model, several limitations exist. Existing research has predominantly utilized data from public databases such as TCGA and GEO. There is a lack of experimental validation regarding the specific functions of these biomarkers in AML. Despite MRGs being effective in patient stratification within both the TCGA and GSE146173 datasets, smaller datasets like GSE24395 may suffer from insufficient statistical power. In the future, CRISPR-Cas9 technology can be used to manipulate target genes in AML cell lines. By combining this with assays such as CCK-8, mitochondrial membrane potential detection, and autophagic flux analysis, we can clarify how these genes regulate mitophagy pathways and cellular phenotypes. The *in vivo* functionality can be verified using AML mouse models. Furthermore, ChIP - seq and proteomics technologies can be utilized to analyze the regulatory network of these biomarkers within the context of AML cell heterogeneity and the immune microenvironment. This analysis can provide theoretical support for developing novel therapies that target mitophagy pathways.

## 5 Conclusion

Five prognostic biomarkers—*ITGB2*, *VIP*, *PTK2*, *FHL2*, and *BAG3*—were identified and integrated into a novel risk model to stratify patients with AML into high- and low-risk groups. This classification revealed distinct molecular and immunological profiles between the two groups, as demonstrated by differences in functional enrichment patterns, immune cell infiltration, somatic mutation landscapes, and drug sensitivity. The findings offer a set of clinically relevant biomarkers with prognostic and therapeutic significance, providing new insights into personalized treatment strategies and disease management in AML.

## Data Availability

The original contributions presented in the study are included in the article/**Supplementary Files**, further inquiries can be directed to the corresponding author/s.
