# Supplementary material for: Identification of the prognostic effect of mitophagy-related genes in acute myeloid leukemia
Source: Front Immunol. 2025 Aug 12;16:1580597. doi: 10.3389/fimmu.2025.1580597 (PMC12378125; doi:10.3389/fimmu.2025.1580597)
Supplement: Supplementary file 3 [file DataSheet3.pdf]

Supplementary Material

1 Supplementary Data

2 Supplementary Figures and Tables

2.1 Supplementary Figures

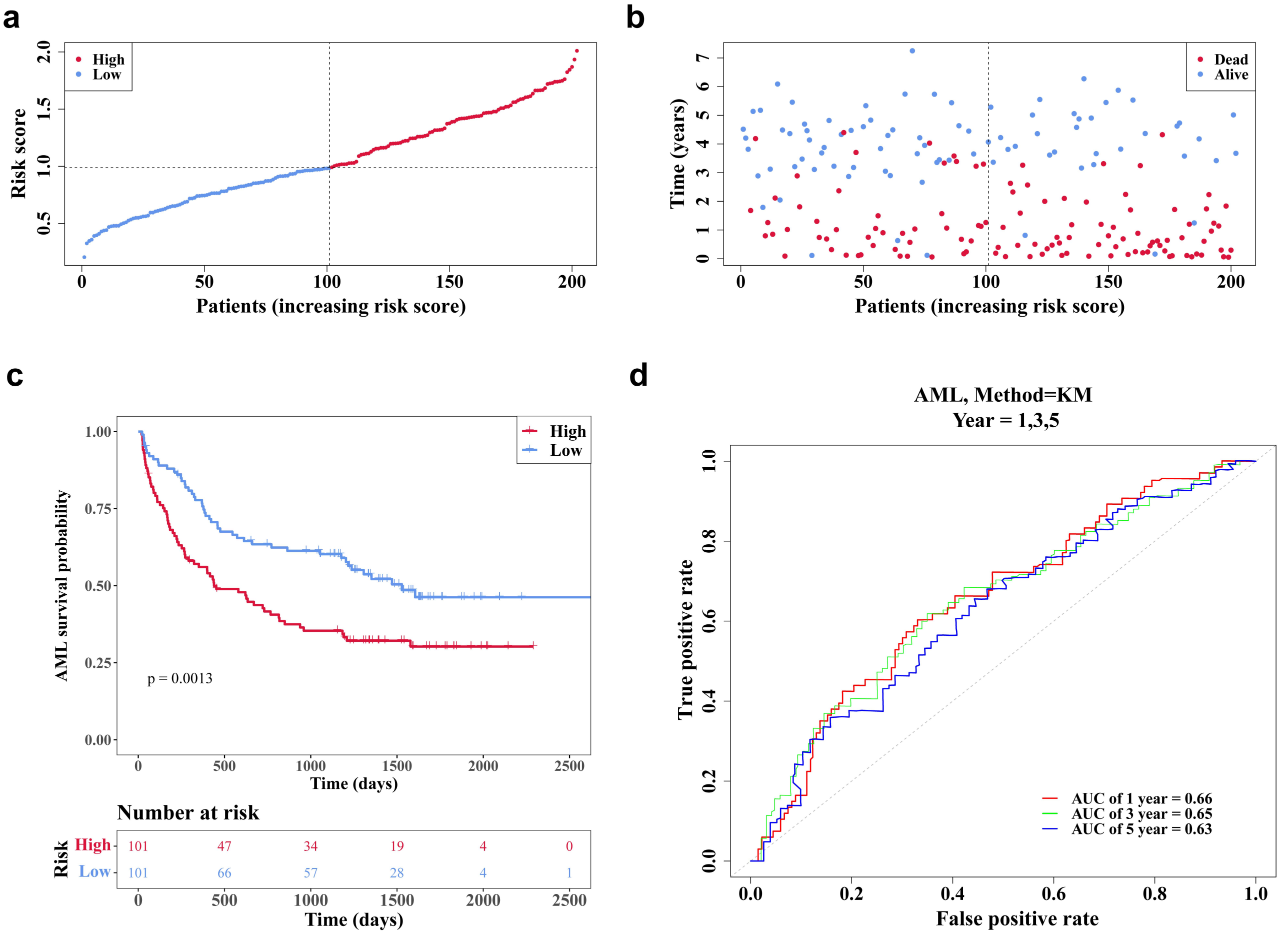

Supplementary Figure 1. Validation of concentration risk models. (a) In the validation set, the risk curve shows that red represents the high-risk group and blue represents the low-risk group. (b) In the survival status plot, red dots represent deceased patients, and blue dots represent surviving patients. (c) The high and low risk group K-M curves for the validation set. (d) The ROC curve of the model in validation set. An AUC value greater than 0.6 indicates that the model has certain predictive performance.

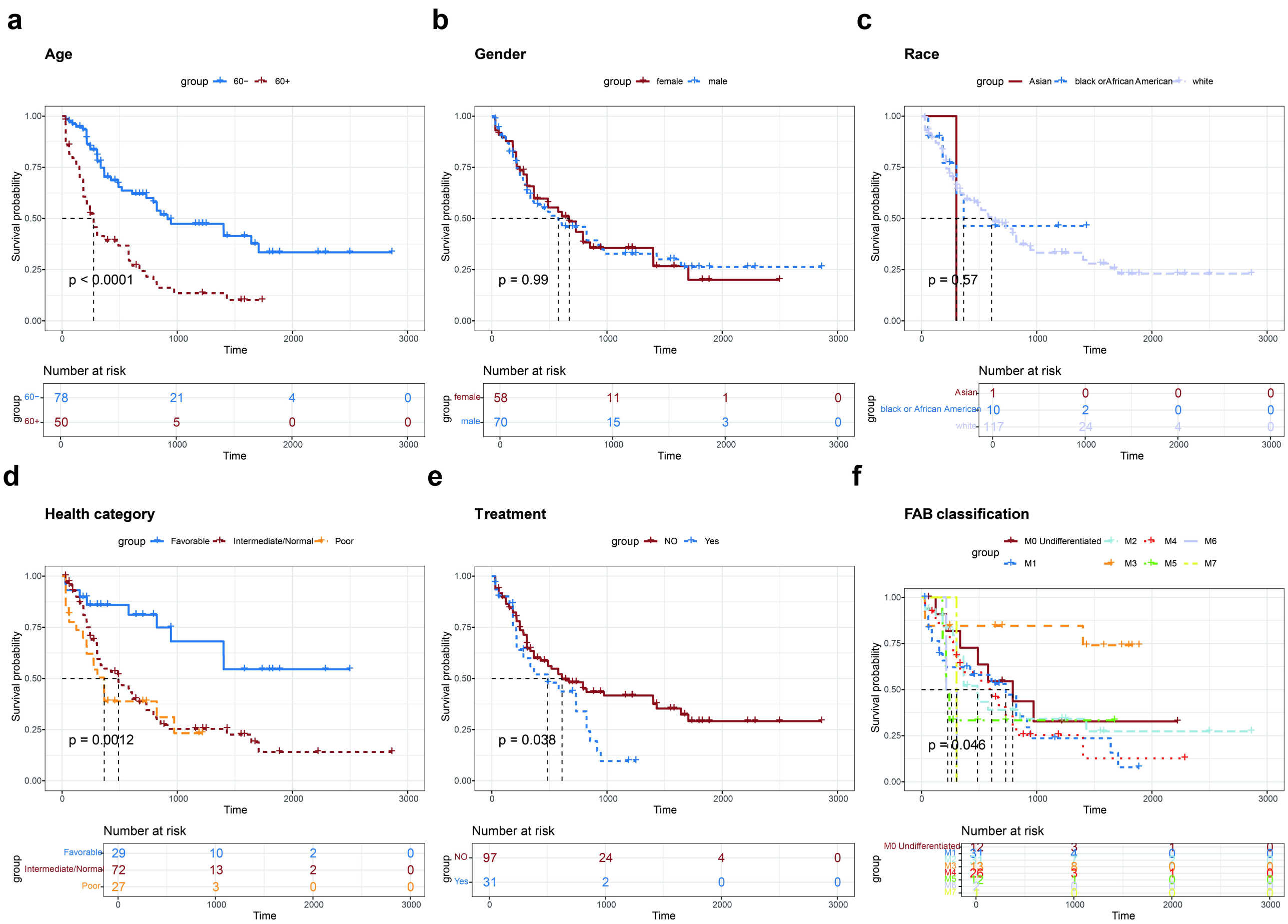

Supplementary Figure 2. Survival analysis among subgroups with different clinical characteristics. (a) K-Mcurves between different age groups. (b) K-M curves between different genders. (c) K-M curves among different race groups. (d) K-M curves among different category groups. (e) K-M curves between different treatment statuses. (f) K-M curves among different FAB classification groups.

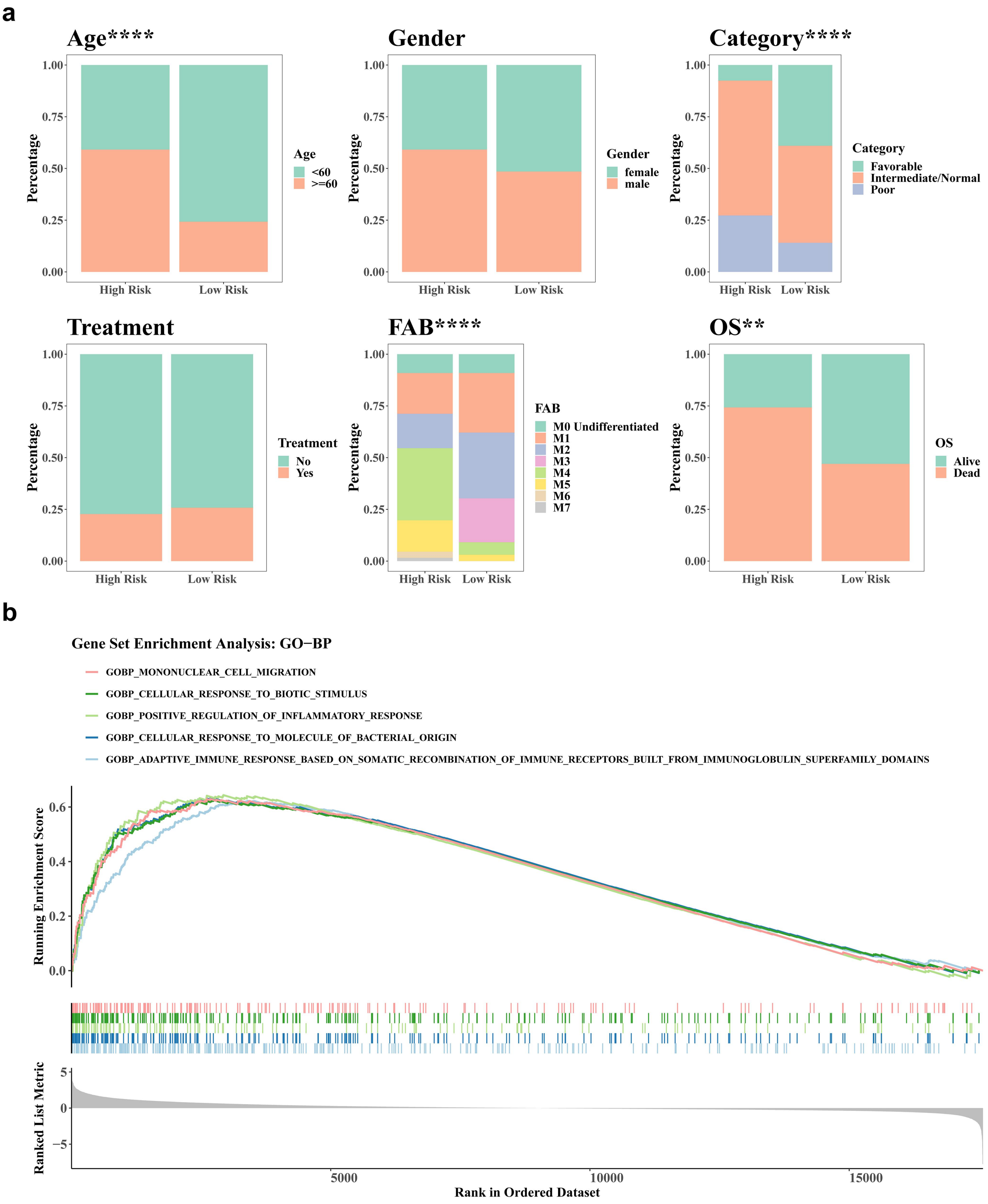

Supplementary Figure 3. Analysis of differences in clinical characteristics among high- and low-risk groups. (a) The differences in risk scores between subgroups with different clinical

characteristics. (b) The GSEA enrichment results for high and low risk groups. *: P < 0.05, **: P< 0.01, ***: P < 0.001, ****: P < 0.0001.

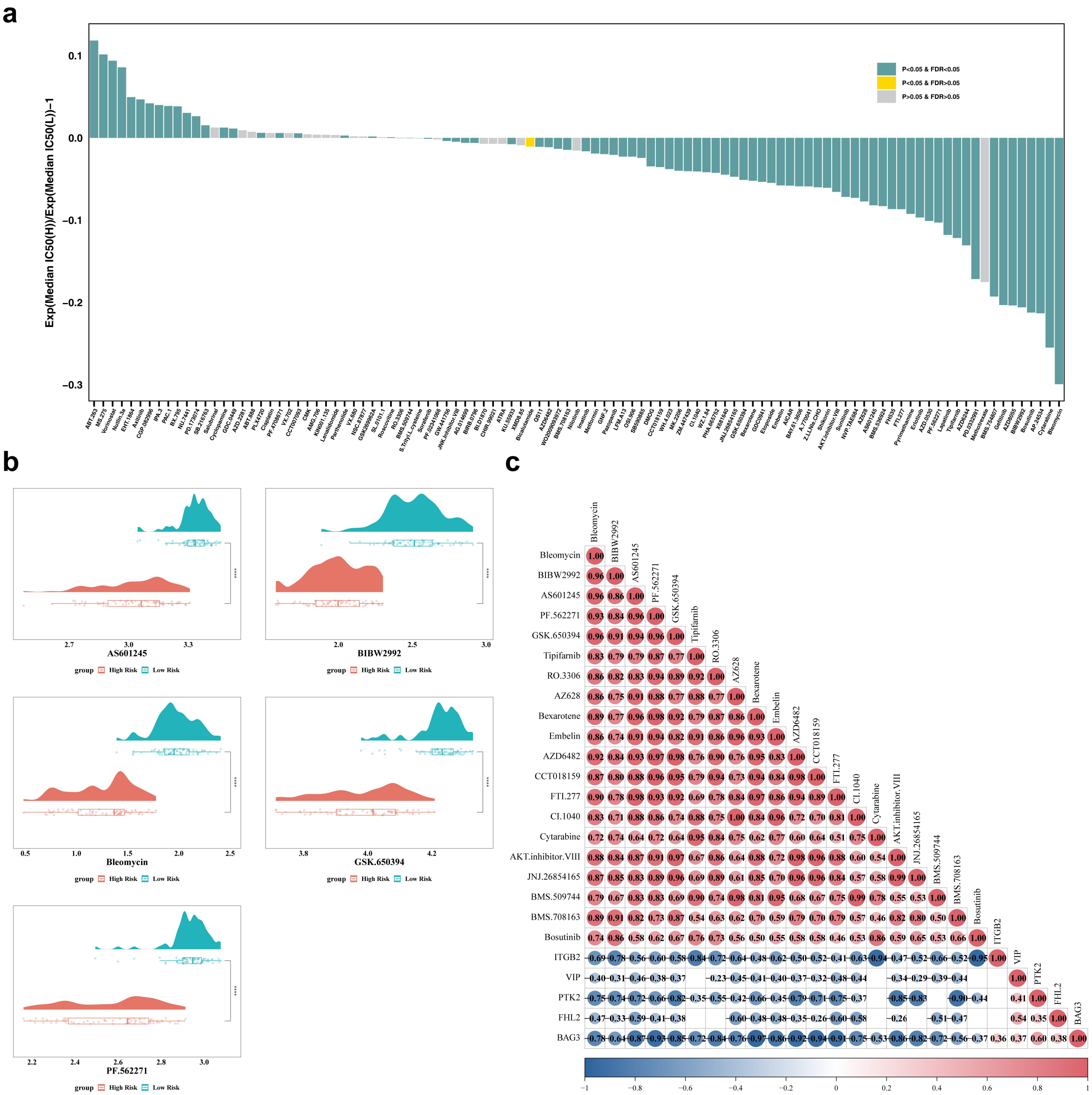

Supplementary Figure 4. (a-b) The differences in IC_{50} of drugs between high and low risk groups. (c) The correlations between the top 20 drugs with significant differences in IC_{50} between high-risk and low-risk groups and five biomarkers

2.2 Supplementary Tables

Supplementary Table 1. A detailed list of 72 MRGs.

Supplementary Table 2. Primer sequences of the genes.

Supplementary Table 3. A detailed list of 26 candidate genes.
