# Supplementary figures and images for: Identification of the prognostic effect of mitophagy-related genes in acute myeloid leukemia
Source: Front Immunol. 2025 Aug 12;16:1580597. doi: 10.3389/fimmu.2025.1580597 (PMC12378125; doi:10.3389/fimmu.2025.1580597)

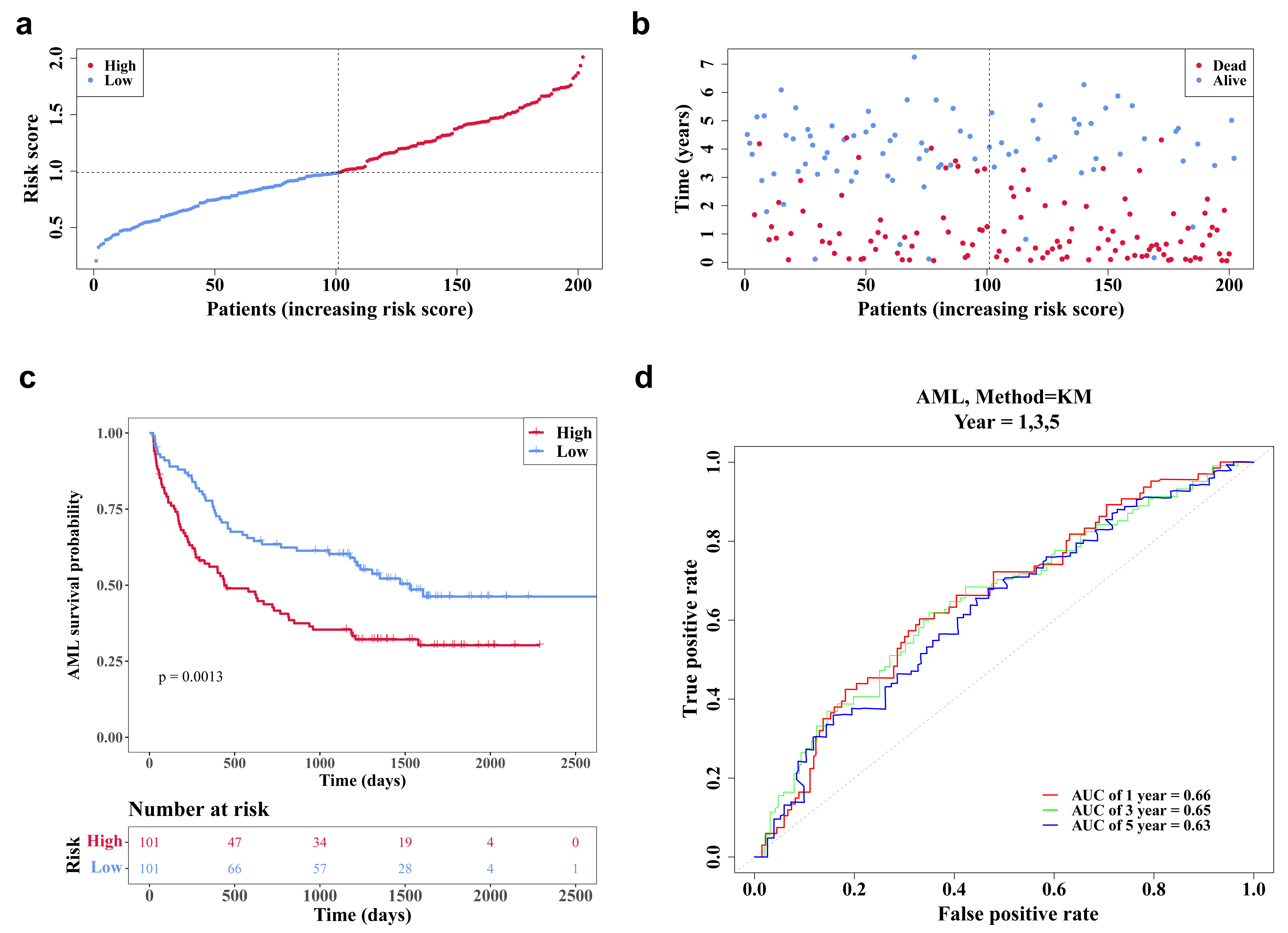

Supplement: Supplementary file 5 [file Image1.tif]

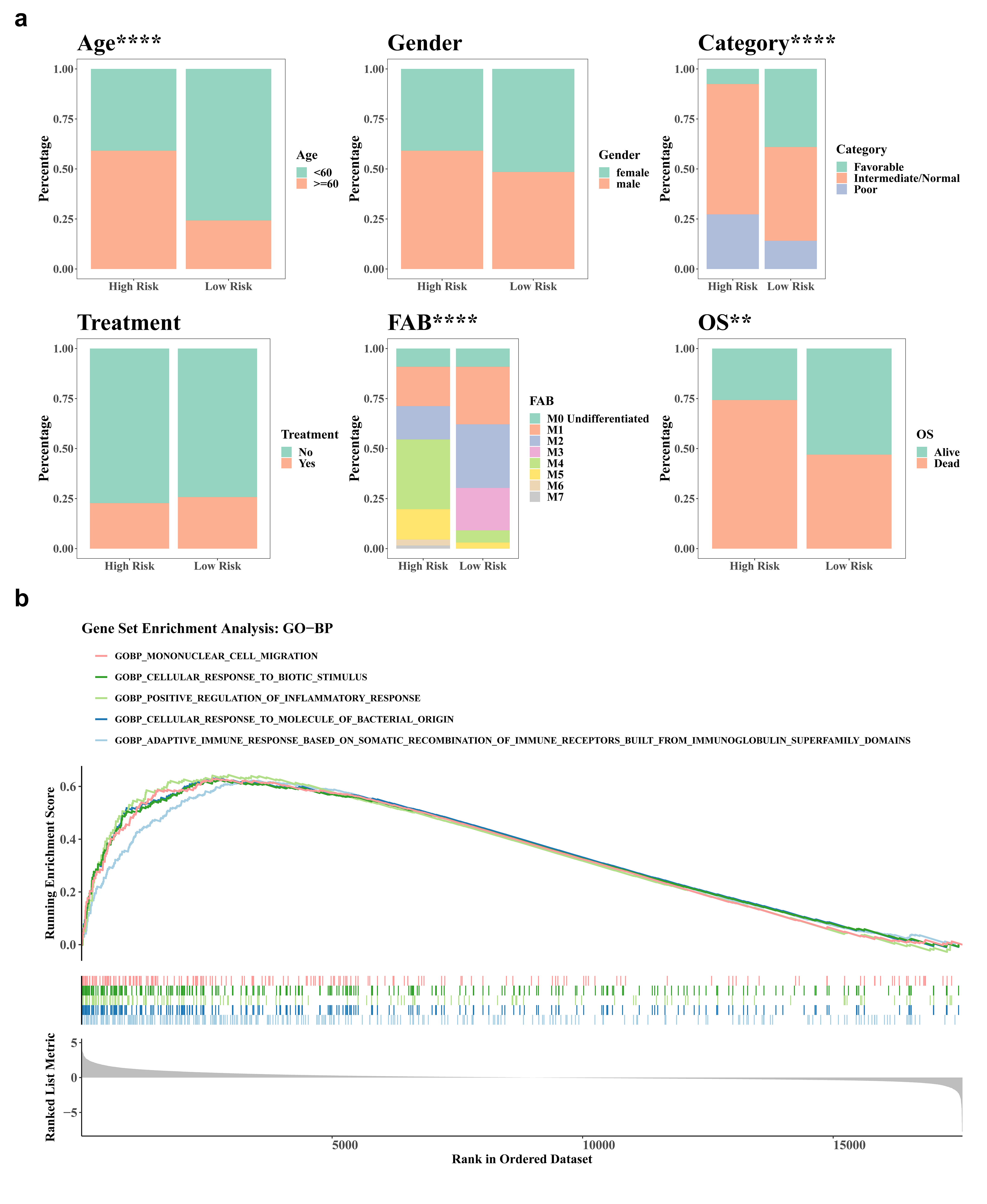

Supplement: Supplementary file 7 [file Image3.tif]
